# The multistate tuberculosis pharmacometric model: a semi-mechanistic pharmacokinetic-pharmacodynamic model for studying drug effects in an acute tuberculosis mouse model

**DOI:** 10.1007/s10928-017-9508-2

**Published:** 2017-02-15

**Authors:** Chunli Chen, Fatima Ortega, Joaquin Rullas, Laura Alameda, Iñigo Angulo-Barturen, Santiago Ferrer, Ulrika SH Simonsson

**Affiliations:** 1 Department of Pharmaceutical Biosciences, Uppsala University, Box 591, 75124 Uppsala, Sweden; 2 Diseases of Developing World Medicines Development Campus, GlaxoSmithKline, Severo Ochoa 2, Tres Cantos, 28760 Madrid, Spain; 3 The Art of Discovery (TAD), Biscay Science and Technology Park, Astondo Bidea, BIC Bizkaia building, No.612, 48160 Derio, Bizkaia, Basque Country Spain

**Keywords:** Mouse, Rifampicin, Tuberculosis, Pharmacokinetics, Pharmacodynamics

## Abstract

The Multistate Tuberculosis Pharmacometric (MTP) model, a pharmacokinetic-pharmacodynamic disease model, has been used to describe the effects of rifampicin on *Mycobacterium tuberculosis* (*M. tuberculosis*) in vitro. The aim of this work was to investigate if the MTP model could be used to describe the rifampicin treatment response in an acute tuberculosis mouse model. Sixty C57BL/6 mice were intratracheally infected with *M. tuberculosis* H37Rv strain on Day 0. Fifteen mice received no treatment and were sacrificed on Days 1, 9 and 18 (5 each day). Twenty-five mice received oral rifampicin (1, 3, 9, 26 or 98 mg·kg^−1^·day^−1^; Days 1–8; 5 each dose level) and were sacrificed on Day 9. Twenty mice received oral rifampicin (30 mg·kg^−1^·day^−1^; up to 8 days) and were sacrificed on Days 2, 3, 4 and 9 (5 each day). The MTP model was linked to a rifampicin population pharmacokinetic model to describe the change in colony forming units (CFU) in the lungs over time. The transfer rates between the different bacterial states were fixed to estimates from in vitro data. The MTP model described well the change in CFU over time after different exposure levels of rifampicin in an acute tuberculosis mouse model. Rifampicin significantly inhibited the growth of fast-multiplying bacteria and stimulated the death of fast- and slow-multiplying bacteria. The data did not support an effect of rifampicin on non-multiplying bacteria possibly due to the short duration of the study. The pharmacometric modelling framework using the MTP model can be used to perform investigations and predictions of the efficacy of anti-tubercular drugs against different bacterial states.

**Electronic supplementary material:**

The online version of this article (doi:10.1007/s10928-017-9508-2) contains supplementary material, which is available to authorized users.

## Introduction

Tuberculosis is an infectious disease caused by *Mycobacterium tuberculosis* (*M. tuberculosis*). Without treatment, mortality rates are high. Rifampicin is an antibiotic discovered in the year of 1957 in the Dow-Lepetit Research Laboratories and used to treat drug-susceptible tuberculosis along with isoniazid, pyrazinamide and ethambutol. The mouse is commonly used as a pre-clinical experimental animal model for evaluating the in vivo efficacy of anti-tubercular compounds, mainly for practical reasons including its size, low associated costs and highly reproducible experimental infections and therapeutic outcomes. The colony forming unit (CFU), a quantification of the bacterial burden in different tissues or fluids [1, 2], is a commonly used pharmacodynamic (PD) endpoint. Bacterial burden is often described using summary PD endpoints such as extent of bacterial killing (e.g. change in CFU at the end of treatment or representative measurements from the bacterial time-kill curves) or rate and duration of bacterial killing (e.g. maximal kill rate or time to reach a certain level of bacteria). These summary PD endpoints do not completely characterise the pharmacokinetics (PK), PD and efficacy over time. The PK/PD indices approach is also commonly used for evaluating the PK and PD of antibiotics and for guiding dose selection [3, 4]. However, the PK/PD indices have several drawbacks as they ignore the time-courses of the PK, PD and bacterial dynamics [5].

The Multistate Tuberculosis Pharmacometric (MTP) model [6] is a semi-mechanistic PK–PD model for studying anti-tubercular drug effects which was developed using in vitro data. It has further been extended to use for clinical data and proven to be able to be used for clinical trial simulations [7]. The model predicts the changes in the numbers of bacteria in the different states (fast-, slow- and non-multiplying bacterial states) with and without drug effects as well as CFU over time. It thus provides a basis for investigating and predicting the effects of anti-tubercular drugs and novel compounds on these bacterial states and CFU. Disease models define the underlying system of the disease and can incorporate the relationships between biomarkers and clinical outcomes, the time course of the disease and potential placebo effects. The parameters of disease models are system-specific, in contrast to those of empirical models where the parameters are both system-specific and drug-specific. A disease model is often linked to a drug model which describes the PK of the drug. Given the disease model and the PK model, the relationship between exposure and response (PK–PD) can be defined. Disease models are available for several therapeutic areas for example malaria [8], myelosuppression [9] and glucose-insulin regulation [10]. The myelosuppression disease model has been shown to be applicable across drugs and various phases of drug development [9, 11]. A similar strategy for tuberculosis drug development using the MTP model would allow the effect on the time-course of CFU due to drug(s) to be predicted in patients from in vivo (animal) and in vitro information.

The aim of this work was to relate rifampicin exposure to the changes in CFU over time in an acute tuberculosis mouse model using the MTP modelling approach.

## Methods

### Chemicals

All chemicals and reagents were obtained from GlaxoSmithKline (GSK). Water was purified and de-ionised using the water purified system. The drugs were orally administered in a solution of Encapsin™ 20% and water 80%.

### Rifampicin pharmacodynamic mouse study

Sixty C57BL/6 mice were anaesthetised with 3% isoflurane (IsoVet®, B.Braun, Piramal Healthcare, Maharashtra, India) and intubated with a metal probe (catalogue number 27134, Unimed SA, Lausanne, Switzerland). Infection was initiated by intratracheal instillation of *M. tuberculosis* H37Rv. The inoculum (10^5^ CFU per mouse suspended in 50 µl of phosphate-buffered saline) was put into the probe and delivered through forced inhalation with a syringe on Day 0. Twenty-five mice received rifampicin (Sigma-Aldrich) 1.00, 2.83, 8.88, 26.4 or 98.0 mg·kg^−1^·day^−1^ orally once daily for 8 days from Day 1 (24 h after infection) and samples were taken after sacrifice on Day 9 after infection. An additional 20 mice were given 30 mg·kg^−1^ rifampicin orally once daily for up to 8 days. Five of these were sacrificed on each of Days 2, 3, 4 and 9 after infection. Fifteen mice received no treatment (natural growth group) and were sacrificed on Days 1, 9 and 18 (five mice on each occasion).

In order to quantify the infection burden in the lungs, all lung lobes were aseptically removed and homogenised. After addition of glycerol (5%), the homogenates were stored frozen (−80 °C) until plating in 10% OADC-7H11 medium for 14 days at 37 °C. After culture, the colonies were counted using an automatic colony counter (a COLyte-Supercount, Synoptics Ltd., Cambridge, United Kingdom), confirmed by visual inspection. The study was ethically reviewed and carried out in accordance with European Directive 2010/63/EU and the GSK Policy on the Care, Welfare and Treatment of Animals.

### The multistate tuberculosis pharmacometric model

**Fig. 1:**
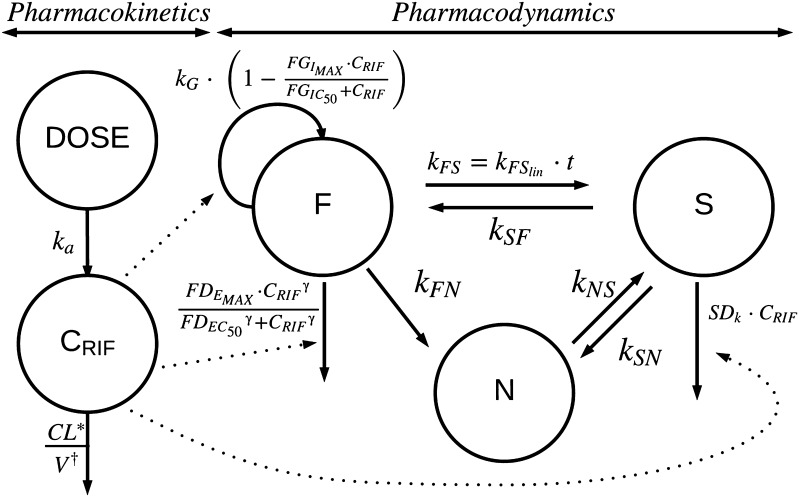
Schematic illustration of the Multistate Tuberculosis Pharmacometric model consisting of fast- (F), slow- (S) and non-multiplying (N) bacterial compartments. The bacterial system was described using the growth rate (*k*_*G*_) of the fast-multiplying bacteria, a time-dependent linear rate parameter $k_{{FS_{lin} }}$, the transfer rate from fast- to slow-multiplying bacterial states (*k*_*FS*_), the first-order transfer rate from slow- to fast-multiplying bacterial states (*k*_*SF*_), the first-order transfer rate from fast- to non-multiplying bacterial states (*k*_*FN*_), the first-order transfer rate from slow- to non-multiplying bacterial states (*k*_*SN*_) and the first-order transfer rate from non- to slow-multiplying bacterial states (*k*_*NS*_). The rifampicin population pharmacokinetic model was a one-compartment model with first-order absorption and elimination accounting for auto-induction. The rifampicin blood concentration (*C*_*RIF*_) was assumed to inhibit the growth of fast-multiplying bacteria and stimulate the death of fast- and slow-multiplying bacteria. $FG_{{I_{MAX} }}$ = the maximum achievable fractional rifampicin-induced inhibition of fast-multiplying bacterial growth rate; $FD_{{E_{MAX} }}$ = the maximum achievable fractional rifampicin-induced stimulation of fast-multiplying bacterial death rate; $FG_{{IC_{50} }}$ and $FD_{{EC_{50} }}$ = the rifampicin concentrations at 50% of $FG_{{I_{MAX} }}$ and $FD_{{E_{MAX} }}$; *γ* = sigmoidicity parameter; *SD*_*k*_ = the second-order slow-multiplying bacterial death rate; *k*_*a*_ = absorption rate constant; *CL* ^*^ = clearance, different between Days 1–2 compared to Days 3–8; *V* ^*†*^ = volume of distribution, different at the lowest dose

A rifampicin population PK model including auto-induction earlier developed in the mouse [12] was linked to the MTP model [6], using a population pharmacokinetic parameter (PPP) approach [13], since PK data was not obtained in this study. This allowed predicting typical rifampicin blood concentrations over time, based on the rifampicin drug regimens applied in this study, as the input to the MTP model. In brief, the population PK model consisted of a one compartment with first-order absorption and elimination (Fig. 1). The volume of distribution at the lowest dose (1.02 mg·kg^−1^) of rifampicin was 2280 and 1250 mL·kg^−1^ for the higher doses. Due to auto-induction of rifampicin, clearance on Days 1 and 2 (79.3 mL·h^−1^·kg^−1^) was statistically significantly lower than that on other days, which was estimated to be 132 mL·h^−1^·kg^−1^. The bioavailability was estimated as 65.6%. The MTP model was simultaneously fitted to all observed CFU (log transformation both sides) versus time data. The MTP model consists of a series of differential equations representing fast-multiplying (F), slow-multiplying (S) and non-multiplying (N) bacterial states (Fig. 1), with first-order linear rate to represent the transfers between states. The estimates of the transfer rates were taken from fitting the MTP model to in vitro data with the same bacterial strain [6], except for the time-dependent transfer rate from fast (F)- to slow (S)-multiplying bacteria (*k*_*FS*_), which was re-estimated using E_MAX_ and linear functions with respect to time in this study (Eqs. 1–2). Re-estimation of *k*_*FS*_ as well as other transfer rates, one at a time, was compared to fixing the parameter to the in vitro estimates [6].

1 $$k_{FS} \; = \;K_{{FS_{lin} }} \times \;t$$

2 $$k_{FS} \; = \;K_{{FS_{sig} }} \times \; \frac{{t_{MAX} \; \times \;t}}{{t_{50} \; + \;t}}$$

where *t* is time; $K_{{FS_{lin} }}$ is the linear increase in *k*_*FS*_ with time; $K_{{FS_{sig} }}$ is the initial transfer rate from F to S; *t*_*MAX*_ is the time to reach the highest value of *k*_*FS*_; and *t*_*50*_ is 50% of *t*_*MAX*_. The growth in CFU in un-treated animals was explored using exponential and Gompertz growth functions. All parameters associated with the natural growth (*k*_*G*_, *k*_*FS*_, *k*_*SF*_, *k*_*FN*_, *k*_*SN*_, *k*_*NS*_) were fixed during the estimation of drug effect. The anti-tubercular effects of rifampicin were evaluated for each possible mechanism in the model, i.e. inhibition of the growth of fast-multiplying bacteria and stimulation of the death of fast-, slow- and non-multiplying bacteria. Different exposure–response relationships were evaluated for each possible mechanism such as linear model, E_MAX_ model, and sigmoidal E_MAX_ model. An exposure–response relationship for each possible effect site was first evaluated alone. Thereafter, all statistical exposure–response relationships were combined and evaluated for statistical significance jointly compared to alone. A final backward evaluation step was also carried out, where the exposure–response relationships were reduced to their simpler forms. A statistically significant criterion of a decrease in the objective function value (OFV) of 3.84, corresponding to a 5% significance level at one degree of freedom was used.

The lower limit of quantification (LLOQ) was 10 CFU/lungs. The M3 method in NONMEM was used to account for PD data below the LLOQ (21.6%). The M3 method, as suggested by Beal et al. [14], is based on simultaneous modelling of continuous and categorical data where the observations below the LLOQ are treated as categorical data. All observations above the LLOQ were estimated using the maximum likelihood estimation method. The likelihoods for observation below the LLOQ were taken to be the likelihood that these observations are indeed LLOQ. Additive and combined error models were evaluated to describe the residual unexplained variability.

### Model selection and evaluation

All modelling was carried out using NONMEM software and FOCE with interaction and Laplace (version 7.3; Icon Development Solutions, Ellicott City, MD, USA) [15]. Model selection was based on the OFV, where a decrease of 3.84 was considered significant (p < 0.05) for one degree of freedom. In addition, scientific plausibility, parameter precision and predictive performance (assessed using a visual predictive check) were used for model selection. In the visual predictive check, 1000 replicates were simulated for the model and 5th, median and 95th percentiles were used with the corresponding data to assess the model performance in Perl-speaks-NONMEM (PsN) (version 4.2.0; Department of Pharmaceutical Biosciences, Uppsala University, Sweden) [16]. Xpose (version 4.4.1; Department of Pharmaceutical Biosciences, Uppsala University, Sweden) [16] was used to visualise the data and results. The run record was produced with Pirana software (version 2.7) [17].

## Results

Simulated typical rifampicin blood concentrations versus time after the different doses at Day 8 after infection are shown in Fig. S1. The final structure of the PK model linked to the MTP model describing the change in CFU over time is shown in Fig. 1. The MTP model bacterial transfer rate constants, except *k*_*FS*_, were fixed in this work to estimates obtained for the same bacterial strain in vitro [6]. Re-estimating the transfer rate from F to S (*k*_*FS*_) as a linear function with time provided a decrease in OFV of 7.3 points, compared to fixing the parameter to the in vitro estimate [6]. Re-estimation of the other transfer rates between the states did not provide a reduction in OFV and was therefore fixed to in vitro estimates [6]. The final model included an exponential growth function for *M. tuberculosis* with an exponential growth rate *k*_*G*_. The initial number (inoculum) of fast-multiplying bacteria in untreated mice (*F*_*0*_^*†*^ *)* differed compared to those in the rifampicin-treated groups (*F*_*0*_). The data did not support inclusion of any inoculum of slow- or non-growing bacteria and these were therefore set to zero in the final model.

The final MTP model included statistically significant and separate rifampicin effects on inhibition of the growth of fast-multiplying bacteria (*I*_*FG*_; Eq. 3), stimulation of the death of fast-multiplying bacteria ($E_{FD}$; Eq. 4) and stimulation of the death of slow-multiplying bacteria (*E*_*SD*_;  Eq. 5). An effect of rifampicin on the non-multiplying bacteria was tested but was not statistically significant and therefore not included in the final model.

3 $$I_{FG} \; = \;\frac{{FG_{{I_{MAX} }} \cdot C_{RIF} }}{{FG_{{IC_{50} }} \; + \;C_{RIF} }}$$

4 $$E_{FD} \; = \;\frac{{FD_{{E_{MAX} }} \cdot C_{RIF}^{\gamma } }}{{FD_{{EC_{50} }}^{\gamma } \; + \;C_{RIF}^{\gamma } }}$$

5 $$E_{SD} \; = \;SD_{k} \cdot C_{RIF}$$

where $FG_{{I_{MAX} }}$ is the maximal achievable fractional rifampicin-induced inhibition of fast-multiplying bacterial growth rate; $FD_{{E_{MAX} }}$ is the maximal achievable fractional rifampicin-induced stimulation of fast-multiplying bacterial death rate; $FG_{{IC_{50} }}$ and $FD_{{EC_{50} }}$ are the rifampicin concentrations at 50% of $FG_{{I_{MAX} }}$ and $FD_{{E_{MAX} }}$, respectively; *SD*_*k*_ is the second-order slow-multiplying bacterial death rate; γ is a sigmoidicity parameter; and *C*_*RIF*_ is the rifampicin blood concentration. The final differential equation systems for fast-multiplying bacteria (F; Eq. 6), slow-multiplying bacteria (S; Eq. 7), and non-multiplying bacteria (N; Eq. 8) changing over time were as follows:

6 $$\frac{dF}{dt}\; = \; k_{G} \cdot \left( {1 - I_{FG} } \right) \cdot F - E_{FD} \cdot F - k_{FS} \cdot F\; + \;k_{SF} \cdot S - k_{FN} \cdot F\;$$

7 $$\frac{dS}{dt}\; = \; k_{FS} \cdot F - k_{SF} \cdot S\; + \;k_{NS} \cdot N - k_{SN} \cdot S - E_{SD} \cdot S$$

8 $$\frac{dN}{dt}\; = \; k_{FN} \cdot F + \;k_{SN} \cdot S - k_{NS} \cdot N$$

where *F*, *S* and *N* represent fast-, slow- and non-multiplying bacteria, respectively. The transfer rates between the bacterial states are given by *k*_*FS*_, *k*_*SF*_, *k*_*FN*_, *k*_*SN*_ and *k*_*NS*_, as shown in Fig. 1.

**Fig. 2:**
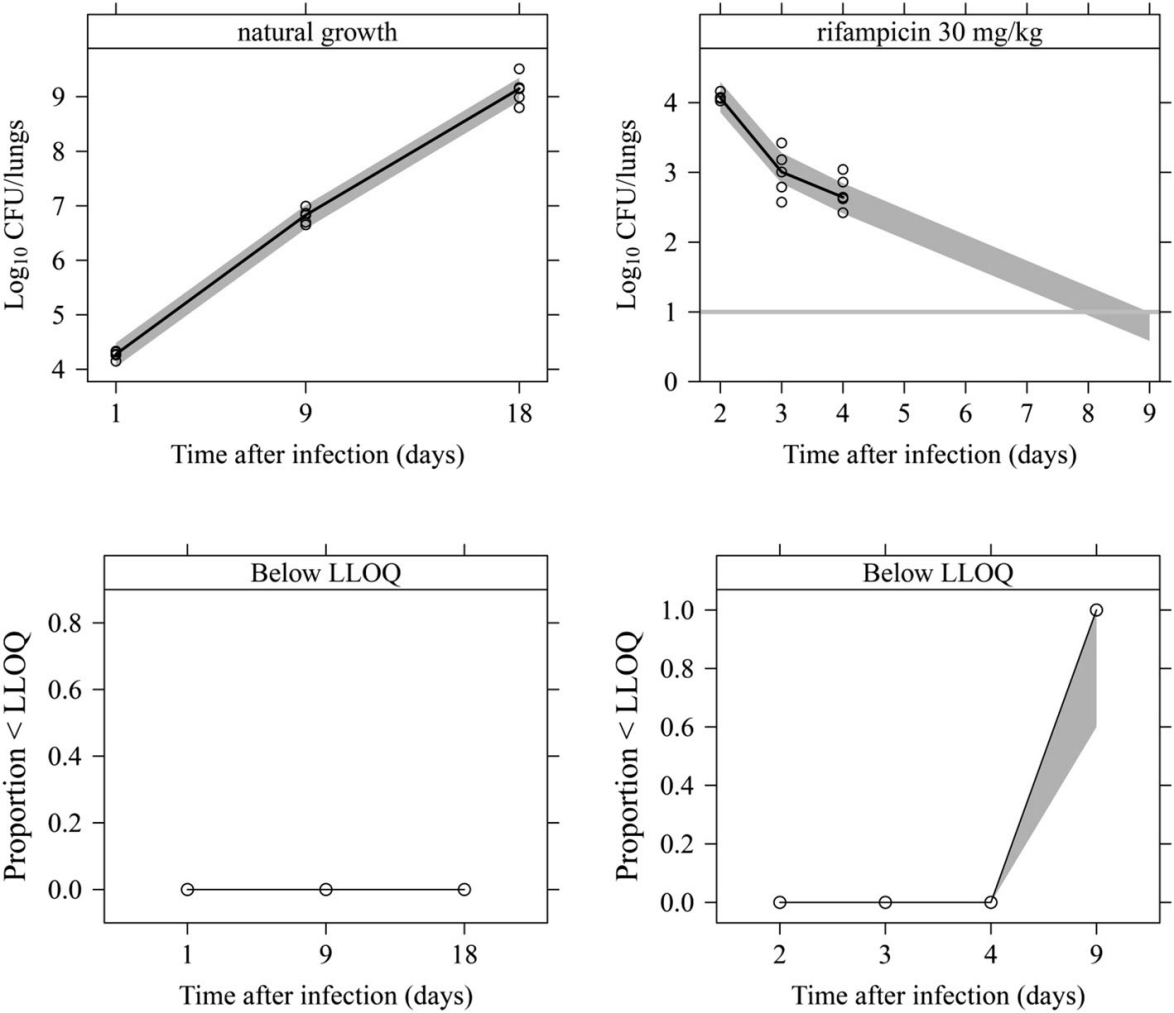
Visual predictive check of the final Multistate Tuberculosis Pharmacometric model applied to colony forming unit (CFU) data from an acute tuberculosis mouse model. In the* left-side* figures, no drug treatment (natural growth) was given and, in the* right-side* figures, rifampicin 30 mg·kg^−1^·day^−1^ (the only dose level with longitudinal observations) was given. The upper and lower figures are from data above and below the lower limit of quantification (LLOQ). In the upper figures, the* solid black line* is the median of the observations. The horizontal* solid grey line* indicates the LLOQ of the CFU data.* Open circles* represent the observations. The* grey shaded* areas represent the 95% confidence intervals for the median of the data simulated by the final model. In the lower plots, the* black solid line* is the median of the data below the LLOQ. The* grey shaded* area is the 95% confidence interval for the median of the data below the LLOQ. There were no data below the LLOQ for the natural growth experiments

**Fig. 3:**
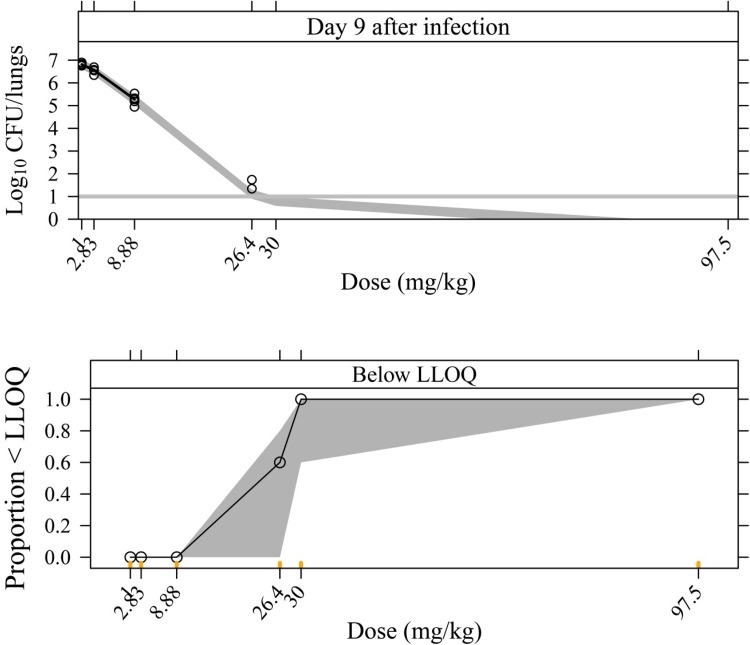
Visual predictive check of the final Multistate Tuberculosis Pharmacometric model applied to colony forming unit (CFU) data using an acute tuberculosis mouse model from dose levels observed only at Day 9 after infection. In the upper figure, log_10_ CFU/lungs declined with increasing doses of rifampicin from 1 to 97.5 mg·kg^−1^·day^−1^ measured at Day 9 after infection. The upper and lower figures are from data above and below the lower limit of quantification (LLOQ), respectively. In the upper figure, the* solid black line* is the median of the observations. No median is shown for the dose of 26.4 mg·kg^−1^ as this dose level only included two observations. The horizontal* solid grey line* indicates the LLOQ of the CFU data.* Open circles* represent the observations. The* grey shaded areas* represent the 95% confidence intervals for the median of the data simulated from the final model. In the lower figure, the *black solid line* is the median of the data below the LLOQ. The* grey shaded area* is the 95% confidence interval for the median of the data below the LLOQ

**Fig. 4:**
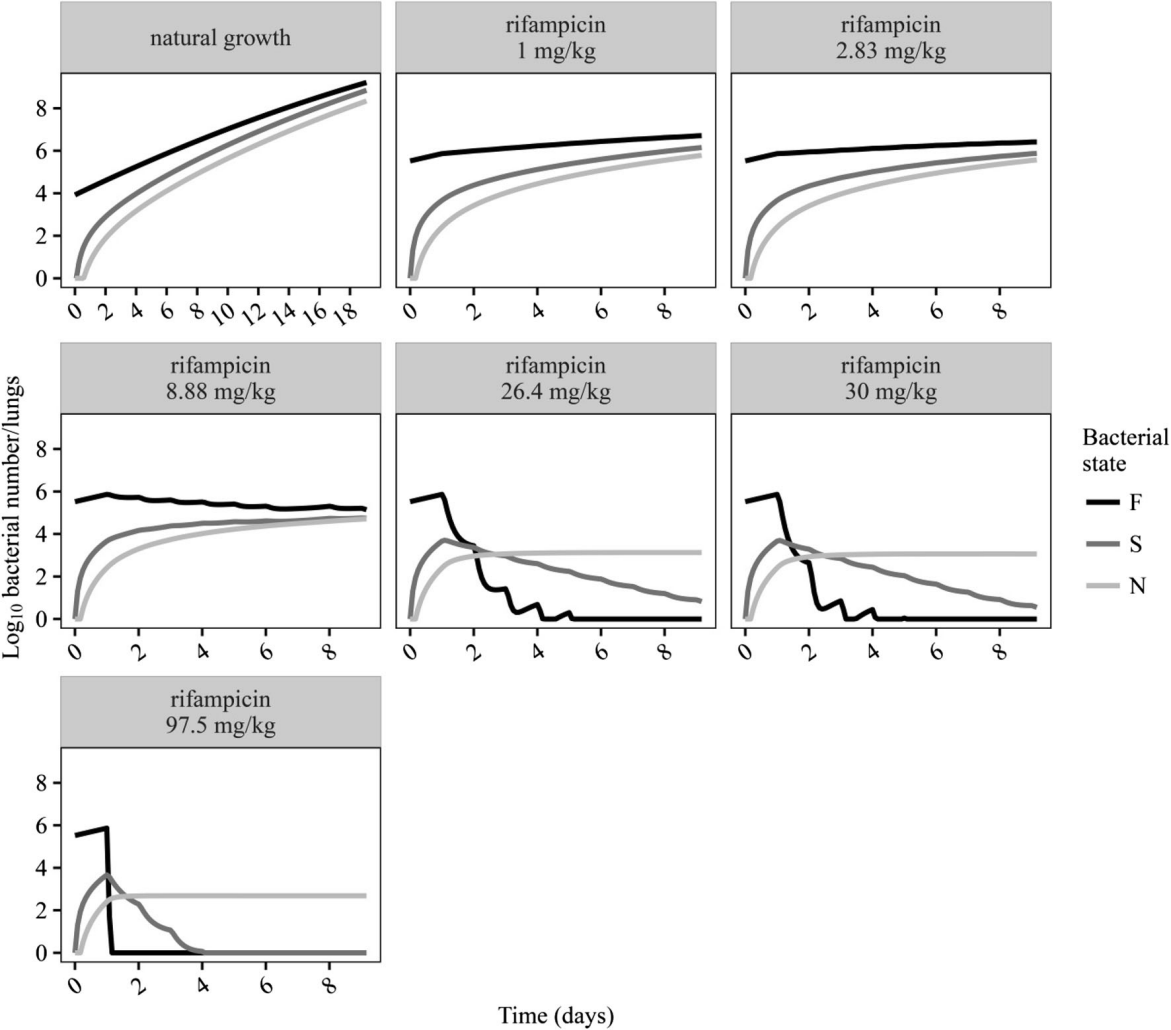
Simulated typical bacterial numbers in the fast- (F), slow- (S) and non-multiplying (N) bacterial states of *M. tuberculosis* using the final Multistate Tuberculosis Pharmacometric model and an acute tuberculosis mouse model without rifampicin treatment (natural growth) and after different dosages of rifampicin. Daily rifampicin treatment began 1 day after the bacterial infection

An additive residual error was used to describe the residual variability using log transformation on both sides. Final parameter estimations for the MTP model are shown in Table 1. The visual predictive check based on the final model for the rifampicin-treated 30 mg·kg^−1^·day^−1^ group and with longitudinal CFU data is shown in Fig. 2. The final model predicted the data well for untreated (natural growth) mice, captured the bi-exponential decline in CFU following rifampicin 30·mg·kg^−1^ over time after infection (Fig. 2) and also described nicely the CFU change after different doses at Day 9 after infection (Fig. 3). The predicted bacterial numbers in the different bacterial states over time without rifampicin treatment (natural growth) and after different rifampicin doses are shown in Fig. 4. The final NONMEM code is available in the supplement S2.

**Table 1:** Final parameter estimates of the Multistate Tuberculosis Pharmacometric (MTP) model for rifampicin in an acute tuberculosis mouse model

Parameter	Description	Typical Value	RSE (%)
F_0_ (lungs^−1^)	Initial number of bacteria in a fast-multiplying bacterial state in treated mice	331000	54.1
F_0_^†^ (lungs^−1^)	Initial number of bacteria in a fast-multiplying bacterial state in untreated mice	8340	8.7
k_G_ (h^−1^)	Growth rate of the fast-multiplying bacteria	0.0333	4.4
${\text{k}}_{{{\text{FS}}_{\text{lin}} }}$ (h^−2^)	Time-dependent transfer rate from fast- to slow-multiplying bacterial states	2.96·10^−5^	29.8
k_SF_ (h^−1^)	First-order transfer rate from slow- to fast-multiplying bacterial states	6.04·10^−4^ FIX^*^	
k_FN_ (h^−1^)	First-order transfer rate from fast- to non-multiplying bacterial states	3.74·10^−8^ FIX^*^	
k_SN_ (h^−1^)	First-order transfer rate from slow- to non-multiplying bacterial states	7.75·10^−3^ FIX^*^	
k_NS_ (h^−1^)	First-order transfer rate from non- to slow-multiplying bacterial states	5.12·10^−5^ FIX^*^	
${\text{FG}}_{{{\text{I}}_{\text{MAX}} }}$	Maximum achievable fractional rifampicin-induced inhibition of fast-multiplying bacterial growth rate	0.716	16.2
${\text{FG}}_{{{\text{IC}}_{50} }}$(µg·mL^−1^)	Rifampicin concentration at 50% of ${\text{FG}}_{{{\text{I}}_{\text{MAX}} }}$	0.0397	50.2
γ	Sigmoidicity parameter	2.19	7.2
${\text{FD}}_{{{\text{E}}_{\text{MAX}} }}$ (h^−1^)	Maximum achievable fractional rifampicin-induced stimulation of fast-multiplying bacterial death rate	352	12.8
${\text{FD}}_{{{\text{EC}}_{50} }}$ (µg·mL^−1^)	Rifampicin concentration at 50% of ${\text{FD}}_{{{\text{E}}_{\text{MAX}} }}$	212	30.4
${\text{SD}}_{\text{k}}$ (mL·h^−1^·μg^−1^)	Second-order slow-multiplying bacterial death rate	4.91·10^−3^	12.9
Error	Additive residual error variability (variance) on log scale	0.217	12.0

FIX^*^: Fixed to estimates from application of the MTP model in vitro [6]

*F* fast-multiplying bacteria; *S* slow-multiplying bacteria; *N* non-multiplying bacteria; *RSE* relative standard error reported on the approximate standard deviation scale

## Discussion

In this study, we describe the application of the MTP model to the CFU data from an acute tuberculosis mouse model. We have shown that the MTP model, which was originally developed using in vitro data [6], can be applied to mouse data by re-estimating the growth function, initial bacterial amounts (*F*_*0*_) and the transfer rate for bacterial transfer from fast- to slow-multiplying bacterial states (*k*_*FS*_). All other parameters in the MTP model, including transfer rates *k*_*SF*_, *k*_*NS*_, *k*_*SN*_ and *k*_*FN*_ were fixed to estimates from applying the MTP model to in vitro data [6]. Linkage of a previously developed rifampicin population PK model [12] to the MTP model allowed the estimation of exposure–response relationships for rifampicin for the different bacterial states in the final model. The final model described the observed CFU versus time profiles well for both natural growth data and data from rifampicin-treated animals (Fig. 2 and Fig. 3). The final model predicted the number of bacteria in the different states over time without rifampicin treatment (natural growth) and after different rifampicin dose levels (Fig. 4). The oscillation in the predicted numbers of fast- and slow-multiplying bacteria was directly related to the PK of rifampicin. Because the data did not support an effect of rifampicin on non-multiplying bacteria, the numbers of non-multiplying bacteria did not oscillate and the increase in non-multiplying bacteria over time was the result of transfer from other bacterial states and the indirect effects of bacterial growth. The growth rate (*k*_*G*_) of fast-multiplying bacteria was best described by an exponential function, because of the lack of plateau CFU data in this mouse model and the short duration of the study. A simplified bacterial growth function like this has been used in the other study [18]. The transfer rate from fast- to slow-multiplying bacteria (*k*_*FS*_) was linearly time dependent within the time range of this study wherefore predictions outside the observed time range cannot be made. The MTP model does not include a natural death rate, based on evidence from an in vitro study [19] showing that the majority of bacilli entered into a viable but non-culturable stage on solid media which was recoverable in the presence of a resuscitation-promoting factor (Rpf). The presence of a large number of Rpf-dependent bacteria was also found in vivo study [19]. These results indicate that the bacilli did not die naturally within the experimental duration of our study, but instead entered into a non-multiplying bacterial state represented by the N-state in the MTP model, which can only be resuscitated by Rpf. We fixed the transfer rate between each bacterial state according to an earlier in vitro study [6], except *k*_*FS*_, which was re-estimated as a linear function of time. This provided a significant drop in OFV (7.3 points), whereas re-estimation of the other rate constants was not statistically significant. The CFU data in this study did not contain enough information about transfer rate from the other bacterial states which was evident in the in vitro data [6]. The final model included rifampicin statistically different drug effects on both an inhibition of the growth and a stimulation of the death of fast-multiplying bacteria which was possible likely due to the cap of the inhibition of the growth through the parameter ${\text{FG}}_{{{\text{I}}_{\text{MAX}} }}$ as well as the very distinct different exposure–response relationships. The EC50s for the two processes were very different (0.04 vs. 212, Table 1). The two exposure–response relationships affecting fast-mutiplying bacterial state i.e. inhibition of the growth and stimulation of the death, were both statistically significant. Removing the drug effect on the inhibition of the growth or stimulation of the death of fast-multiplying bacteria led to an increase in OFV by 9 and 211 points, respectively, indicating that two separate processes of drug effect were described. Omitting the drug effect on the stimulation of the death of slow-multiplying bacteria led to an increase in OFV by 15 points i.e. the drug effect on the slow-multiplying bacterial state was also statistically significant. The CFU count is a sum of the total number of bacteria that are able to multiply on solid media and as such, the CFU assay is not possible to directly distinguish between fast- and slow-multiplying bacterial states. It is hence not possible to assign a specific number of CFU to either the fast- or the slow-multiplying bacterial state. Instead the model was allowed to estimate the number of bacteria that belongs to the fast-multiplying and the slow-multiplying bacterial state. The numbers in the fast- and the slow- multiplying states, as a sum, were part of the model prediction of the CFU data. A similar approach of performing multiple states predictions based on one observation has previously been utilized to describe the life cycle of *Plasmodium falciparum* [8] and *Streptococus pyogenes* [20].

Rifampicin is effective against both replicating and non-replicating bacteria because it targets essential and central DNA machinery [21]. It is thought to inhibit the bacterial DNA-dependent RNA polymerase [22]. In the final MTP model, which incorporates fast-, slow- and non-multiplying bacterial states, rifampicin significantly inhibited the growth of fast-multiplying bacteria and stimulated the death of fast- and slow-multiplying bacteria. Two separate exposure–response relationships were identified for the rifampicin effect on the stimulation of the death of fast-multiplying and slow-multiplying bacterial states described as non-linear and linear relationships, respectively. The final model did not include a rifampicin effect on non-multiplying bacteria as it was not statistically significant. This was possibly due to that the non-growing subpopulation was a minority segment of the total bacterial burden in this acute tuberculosis mouse model. The MTP model did not include any immune response element as it was based on in vitro time-kill experiments lacking immune response. However, an immune response may occur in an in vivo system such as that represented by the mouse. The MTP model could be extended to include immune response components. However, it was not considered necessary for this study, since the adaptive immune response under similar experimental conditions [23] did not impair the growth of *M. tuberculosis* during the assay period in C57BL/6 mice. As such, there was no effect of the immune system on the CFU versus time relationship in this study.

A limitation in this study was that PK information was not collected from the mouse that provided the PD information instead a PPP approach [13] was used. The population PK model applied in this study was developed using healthy animals [12], and we assumed that the PK would be similar in the *M. tuberculosis*-infected animals used in the PD experiments. The PK in infected animals, especially in the acute infection setting, can be quite different compared to healthy animals. In addition, the earlier developed population PK model was built using data of only 3 days of rifampicin administration, not fully capturing the auto-induction process whereas in the current experiment animals, rifampicin was dosed up to 8 days. In this study, a wide dose range (1–98 mg·kg^−1^) was studied. The applied population PK model was built on a dose range of rifampicin from 1 to 100 mg·kg^−1^, which is covering the dose range in this study where it was shown earlier that rifampicin PK is dose-dependent in this range. A PPP approach ignores inter-animal variability in exposure and is therefore less informative than the use of PK information from the animal in which the PD response was obtained. However, the inter-animal variability is likely to be low but potential differences between healthy and diseased animals (covariate effect) are not known.

## Conclusions

The MTP model described the changes in CFU over time well in an acute tuberculosis mouse model and rifampicin treatment effects on different bacterial states were characterised. The pharmacometric modelling framework using the MTP model can be used to perform investigations and predictions of the efficacy of anti-tubercular drugs against different bacterial states.

## Electronic supplementary material

Below is the link to the electronic supplementary material.

Simulated typical concentrations versus time after the different doses at Day 8 after infection in mice. Supplementary material 1 (DOCX 672 kb)

Supplementary material 2 (DOCX 43 kb)
