# Supplementary figures and images for: The multistate tuberculosis pharmacometric model: a semi-mechanistic pharmacokinetic-pharmacodynamic model for studying drug effects in an acute tuberculosis mouse model
Source: J Pharmacokinet Pharmacodyn. 2017 Feb 15;44(2):133–41. doi: 10.1007/s10928-017-9508-2 (PMC5376397; doi:10.1007/s10928-017-9508-2)

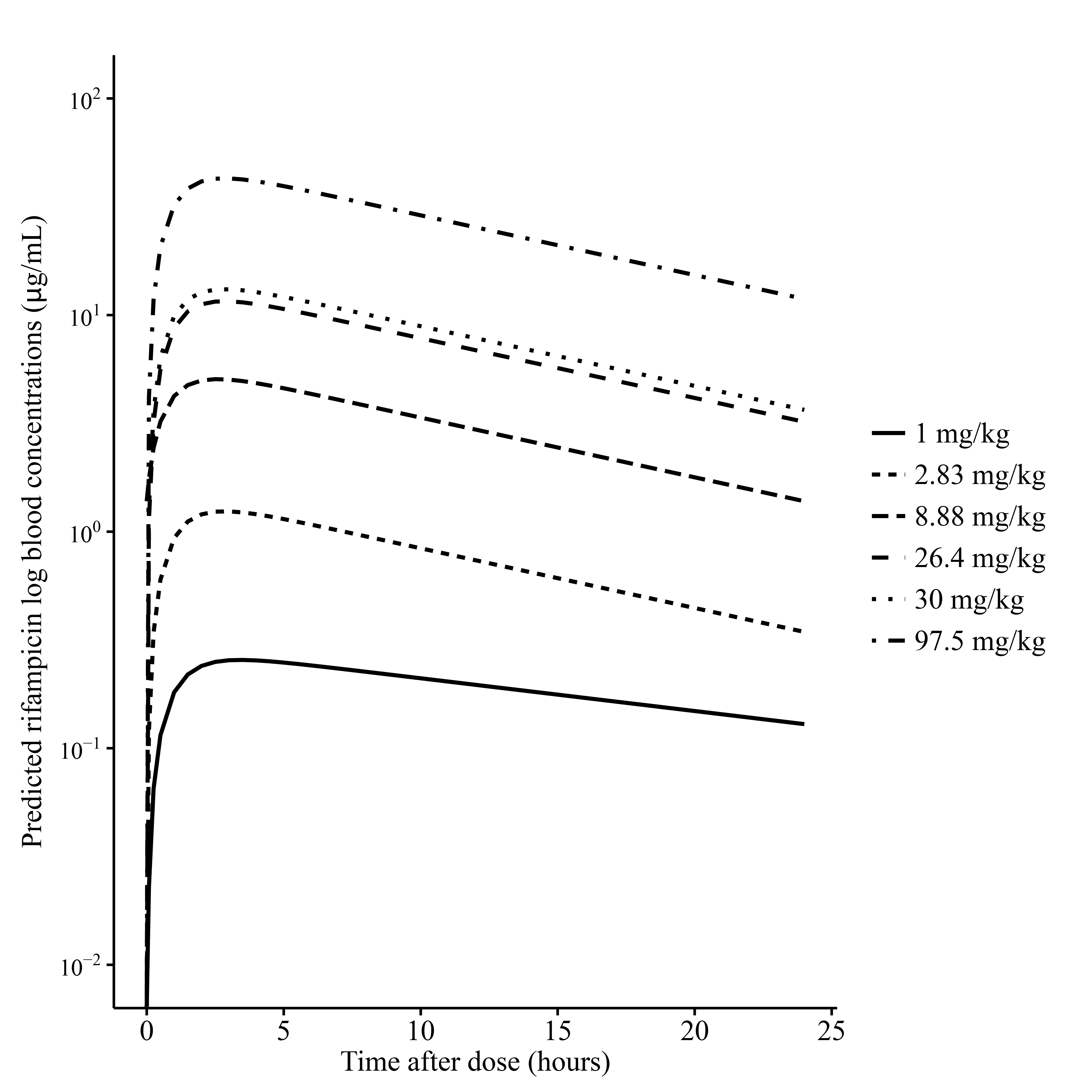

Supplement: Supplementary file 1 — Simulated typical concentrations versus time after the different doses at Day 8 after infection in mice. Supplementary material 1 (DOCX 672 kb) [file 10928_2017_9508_MOESM1_ESM.docx]
