# Supplementary material for: The multistate tuberculosis pharmacometric model: a semi-mechanistic pharmacokinetic-pharmacodynamic model for studying drug effects in an acute tuberculosis mouse model
Source: J Pharmacokinet Pharmacodyn. 2017 Feb 15;44(2):133–41. doi: 10.1007/s10928-017-9508-2 (PMC5376397; doi:10.1007/s10928-017-9508-2)
Supplement: Supplementary file 2 — Supplementary material 2 (DOCX 43 kb) [file 10928_2017_9508_MOESM2_ESM.docx]

| **Article title** | The Multistate Tuberculosis Pharmacometric Model – A Semi-Mechanistic Pharmacokinetic-Pharmacodynamic Model for Studying Drug Effects in an Acute Tuberculosis Mouse Model |
| --- | --- |
| **Journal name** | Journal of Pharmacokinetics and Pharmacodynamics |
| **Author names** | Chunli Chen^1,*^, Fatima Ortega^2^, Joaquin Rullas-Trincado^2^, Laura Alameda^2^, Iñigo Angulo-Barturen^2, 3^, Santiago Ferrer^2^, Ulrika SH Simonsson^1^ |
| **Author affiliations** | ^1^Department of Pharmaceutical Biosciences, Uppsala University, P.O. Box 591, 751 24 Uppsala, Sweden  ^2^Diseases of Developing World Medicines Development Campus, GlaxoSmithKline, Severo Ochoa 2, 28760, Tres Cantos, Madrid, Spain  ^3^The Art of Discovery (TAD), Biscay Science and Technology Park, BIC Bizkaia, Astondo, 48160 Derio, Bizkaia, Basque Country, Spain |
| **^*^Corresponding author** | Chunli Chen : chunli.chen@farmbio.uu.se |

**Caption**: NONMEM model code for the Combined PK model and Final Multistate Tuberculosis Pharmacometric (MTP) model applied to mouse data

$PROBLEM Combined PK and MTP model in the mouse

$INPUT ID TIME DRUG EVID DV DOSE AMT WT FLAG TYPE

; FLAG (0=dose, 1= PK obs, 2= PD obs)

; TYPE (0=dose, 1= PK obs, 2= PD obs> LLOQ, 3= PD obs ≤ LLOQ)

$DATA rif_pkpd.csv IGNORE=@

$SUBROUTINE ADVAN13 TOL=9

$MODEL

COMP = (RIF,DEFDOSE) ; rifampicin dose cmt

COMP = (CENTRAL) ; rifampicin central PK compartment

COMP = (F) ; fast-multiplying bacteria

COMP = (S) ; slow-multiplying bacteria

COMP = (N) ; non-multiplying bacteria

$PK

; pharmacokinetic model from Chen et al. Eur J Pharm Sci 2016

TVCL = THETA(1)

IF(TIME.GE.48) TVCL = THETA(2)

TVV = THETA(3)

IF(DOSE.LE.1020) TVV = THETA(4)

CL = TVCL

V = TVV

KE = CL/V

TVKA = KE + THETA(5)

TVF1 = THETA(6)

IF(DOSE.EQ.0) TVF1 = 0

S2 = V

KA = TVKA

F1 = TVF1

F2 = 1

; Multistate Tuberculosis Pharmacometric (MTP) model

TVAF = THETA(7)*EXP(ETA(1))

IF(DRUG.EQ.0) TVAF = THETA(8)

TVKGR = THETA(9)

TVKFSLIN = THETA(10)/100000

TVKFN = THETA(11)/1000000

TVKSF = THETA(12)/10

TVKSN = THETA(13)

TVKNS = THETA(14)/100

TVFGIMAX = THETA(15)

TVFGIC50 = THETA(16)

TVFDGAM = THETA(17)

TVFDEMAX = THETA(18)

TVFDEC50 = THETA(19)

TVSDK = THETA(20)

AF = TVAF

KGR = TVKGR

KFSLIN = TVKFSLIN

KSF = TVKSF

KFN = TVKFN

KSN = TVKSN

KNS = TVKNS

FGIMAX = TVFGIMAX

FGIC50 = TVFGIC50

FDGAM = TVFDGAM

FDEMAX = TVFDEMAX

FDEC50 = TVFDEC50

SDK = TVSDK

A_0(1) = 0.00001

A_0(2) = 0.00001

A_0(3) = AF

A_0(4) = 0.00001

A_0(5) = 0.00001

$DES

RIFCB = A(2)/V ; rifampicin blood concentration

IF(DRUG.EQ.0) RIFCB = 0

; exposure-response relationships for the MTP model

FD = (FDEMAX*RIFCB**FDGAM)/(FDEC50**FDGAM+RIFCB**FDGAM)

SD = SDK*RIFCB

FG = 1-((FGIMAX*RIFCB)/(FGIC50+RIFCB))

; time-dependent transfer rate in the MTP model

KFS = KFSLIN*T

DADT(1) = -KA*A(1)

DADT(2) = KA*A(1)-KE*A(2)

DADT(3) = KGR*A(3)*FG-KFS*A(3)+KSF*A(4)-KFN*A(3)-FD*A(3)

DADT(4) = KFS*A(3)-KSF*A(4)-KSN*A(4)+KNS*A(5)-SD*A(4)

DADT(5) = KFN*A(3)+KSN*A(4)-KNS*A(5)

$ERROR

COM2 = 0

IF(FLAG.EQ.1) COM2 = 1

COM3 = 0

IF(FLAG.EQ.2) COM3 = 1

DEL = 1E-6

IF(AMT.GT.0) RTAD = TIME

TAD = TIME-RTAD

IF(FLAG.EQ.1) THEN

IPRED = A(2)/V

IRES = DV - IPRED

PROP = IPRED*SQRT(SIGMA(1,1))

IWRES = IRES/(PROP+0.000001)

Y1 = IPRED+IPRED*EPS(1)

ENDIF

FBAC = A(3)

SBAC = A(4)

NBAC = A(5)

TBAC = A(3)+A(4)+A(5)

IPRED = LOG(A(3)+A(4))

SIG = SQRT(SIGMA(2,2))

IRES = DV - IPRED

IWRES = IRES/(SIG+DEL)

LLOQ = LOG(10)

DUM = (LLOQ-IPRED)/SIG

CUMD = PHI(DUM)

IF(FLAG.EQ.2.AND.TYPE.EQ.2) THEN

F_FLAG = 0

Y2 = IPRED + EPS(2)

ELSE

F_FLAG = 1

Y2 = CUMD

END IF

Y = COM2*Y1 + COM3*Y2

$THETA

; Population PK parameters

79.321 FIX ; 1.CL at Days≤ 2

131.911 FIX ; 2.CL at Days ≥3

1249.140 FIX ; 3.V at Dose > 1020 ug/kg

2278.740 FIX ; 4.V at Dose ≤1020 ug/kg

0.994 FIX ; 5.KA

0.656 FIX ; 6.F1

; Natural growth parameters

; Fixed parameters from in vitro work of Clewe et al J Antimicrob Chemother 2016

(0,332089.0) ; 7.F inoculum of treated mice

(0,8348.540) ; 8.F inoculum of untreated mice

(0,0.033255) ; 9.KGR

(0,2.956210) ; 10.KFSLIN

0.037375 FIX ; 11.KFN (in vitro: 0.897x10^-6^, 0.897x10^-6^/24)

0.006042 FIX ; 12.KSF (in vitro: 0.0145, 0.0145/24)

0.007750 FIX ; 13.KSN (in vitro: 0.186, 0.186/24)

0.005125 FIX ; 14.KNS (in vitro: 0.123x10^-2^, 0.123x10^-2^/24)

; Exposure response parameters

(0,0.717058,1) ; 15.FGIMAX

(0,0.039927) ; 16.FGIC50

(0,2.192450) ; 17.FDGAM

(0,67.08230,600) ; 18.FDEMAX

(0,98.50150,300) ; 19.FDEC50

(0,0.004911) ; 20.SDK

$OMEGA 0 FIX

$SIGMA 0.0235 FIX

$SIGMA 0.216544

$ESTIMATION METHOD=1 LAPLACIAN INTER PRINT=3 MAXEVAL=9999 NSIG=3 SIGL=9 NOABORT

$COVARIANCE

$TABLE ID TIME DV PRED IPRED DOSE FLAG TYPE NOPRINT ONEHEADER FILE=sdtab1

$TABLE ID TIME AF KGR KFSLIN FGIMAX FGIC50 FDGAM FDEMAX FDEC50 SDK FG FD SD FBAC SBAC NBAC NOPRINT ONEHEADER FILE=patab1
